# A Semi-Automated Single Day Image Differencing Technique to Identify Animals in Aerial Imagery

**DOI:** 10.1371/journal.pone.0085239

**Published:** 2014-01-14

**Authors:** Pat Terletzky, Robert Douglas Ramsey

**Affiliations:** Department of Wildland Resources, Utah State University, Logan, Utah, United States of America; University of Rome ‘La Sapienza’, Italy

## Abstract

Our research presents a proof-of-concept that explores a new and innovative method to identify large animals in aerial imagery with single day image differencing. We acquired two aerial images of eight fenced pastures and conducted a principal component analysis of each image. We then subtracted the first principal component of the two pasture images followed by heuristic thresholding to generate polygons. The number of polygons represented the number of potential cattle (*Bos taurus*) and horses (*Equus caballus)* in the pasture. The process was considered semi-automated because we were not able to automate the identification of spatial or spectral thresholding values. Imagery was acquired concurrently with ground counts of animal numbers. Across the eight pastures, 82% of the animals were correctly identified, mean percent commission was 53%, and mean percent omission was 18%. The high commission error was due to small mis-alignments generated from image-to-image registration, misidentified shadows, and grouping behavior of animals. The high probability of correctly identifying animals suggests short time interval image differencing could provide a new technique to enumerate wild ungulates occupying grassland ecosystems, especially in isolated or difficult to access areas. To our knowledge, this was the first attempt to use standard change detection techniques to identify and enumerate large ungulates.

## Introduction

Aerial imagery has been used to manually count and estimate population abundances of a diverse array of wildlife species from birds [1]–[4] to terrestrial species [5]–[6] to marine mammals [7]–[11]. Sources of variation in animal enumeration include observers, habitat or vegetative cover, topography, and animal behavior. For example, manual counting of birds in photographs has resulted in inconsistent counts both within and among observers [1], while counts were less variable for larger bodied terrestrial and marine mammals in homogenous habitats [5]–[13]. Terrestrial systems with little vegetative structure, such as grasslands or tundra, had fewer features that caused confusion when enumerating individual animals and were usually correlated with lower variation in counts. Manual counts from aerial photographs of buffalo (*Syncerus caffer*) and wildebeest (*Connochaetes taurinus albojubatus*) in the Serengeti grasslands resulted in consistent counts across four years [14]. In the Arctic tundra, which has little vegetative structure and is relatively homogenous, two independent surveyors counted similar numbers of caribou (*Rangifer tarandus*) [15].

The importance of background homogeneity was also influential in the detection of deer (*Odocoileus* spp.) in arid uplands [16] and detection was affected by the amount of dried brush in the background [17]. Sandy beaches, non-vegetated islands, ice flows or even the ocean itself are uniform backgrounds that provide easy differentiation between an animal and its surroundings. The rocky shores and beaches used by sea lions (*Zalophus californianus*) provided a consistent background in which the number of pups that were manually counted from aerial photographs was similar to the count obtained from ground surveys [12], [13]. These studies suggest that large-bodied mammals can be counted in aerial photographs especially in habitats that have simple backgrounds from which they can readily be differentiated [18], [19].

Temporal change detection from remotely sensed imagery via image subtraction has been used to quantify changes in land cover, habitat types, forests species composition, landscape health (i.e., flooding, landslides, drought), and when mapping urban growth [20], [21]. The temporal scales used to detect change have ranged from seasonal to decadal [22]–[25] and frequently focused on the detection or differentiation of change versus no-change. Change detection at finer temporal scales, such as within a month, a week, or a day are generally derived by subtracting spatially registered frames derived from airborne digital camera or video systems [26]. Theoretically, image differencing at such fine temporal scales should differentiate moving features such as animals by quantifying the change in the spectral responses of pixels with and without animal features [27].

One advantage of airborne or satellite imagery is the permanent, unaltered record of conditions across a landscape at a single period in time. The imagery will record, in perpetuity, features present on the landscape, allowing for exploratory and innovative analyses of the imagery without change to the source data. A second advantage of remotely sensed imagery is the possibility of acquiring spectral information undetectable by human vision, such as infrared or thermal data, to describe and identify features. A third advantage is that remotely acquired imagery has the potential to reduce or eliminate negative responses of animals to low flying aircraft [28]–[32]. Finally, remotely sensed imagery can be readily obtained over isolated or difficult to reach areas (e.g., the Mongolian steppe, parts of the South African continent). The advantages of remotely sensed imagery have been recognized in numerous fields resulting in a significant increase in the amount of imagery collected by various platforms in the past few years. Concomitant with the increase in the volume of imagery is the need for automated analysis to extract information from imagery. Manual evaluation of aerial or satellite imagery is time consuming, subjective and thus not repeatable. Automated or semi-automated analysis of remotely sensed imagery has been shown to reduce workload, increase efficiency, and improve operator performance [33], [34].

We examined a semi-automated technique that employed single day image differencing to enumerate cattle and horses in fenced pastures. We theorized that pixels representing animals in the first image would have a different spectral response than those same pixels in the second image, due to animals moving out of the pixel area (change). We further theorized that pixels not representing animals (i.e., vegetation), would exhibit similar spectral values between the first and second images (no change). Fenced pastures provided a convenient test case as the number of animals in a pasture did not alter between image acquisitions and a definitive number of individuals in a pasture could be determined from ground counts or verbal confirmation obtained from landowners.

## Materials and Methods

### Study Locations

On October 31, 2006, we acquired multispectral airborne imagery from a fixed wing aircraft under mostly clear skies across portions of Cache Valley (CV) and a portion of Box Elder County west of Brigham City (BC) in northern Utah. Cache Valley is a north-south trending valley with a mean annual precipitation of 45 cm [35] and an elevation of 1,355 m [36] at the center of the valley. CV sites were located in the valley bottomlands dominated by a mixture of dense and sparse grasslands. Brigham City (BC) is located in the Basin and Range physiographic province and sits on the western base of the north-trending Wellsville Mountains. The BC sites had a mean precipitation of 47 cm [35] with a mean elevation of 1,289 m [36] and were dominated by sparse grasslands.

### Animal Ground Counts

Rather than compare one estimate to another estimate, we were able to compare the number of animals identified by image differencing to the known number of animals in each pasture. Ground enumeration of domestic cattle and horses occurred at the same time as image acquisition. Counting was within 300 m of the animals and without physical obstructions such as large hills or trees. When possible, we contacted landowners to corroborate ground counts. As group size increased beyond 50 animals, accurate enumeration of individuals lessened due to animals moving around which resulting in possible replicate counts or missing an individual entirely [19], [37], [38]. Although the accuracy was not determined for the ground counts, by limiting analysis to those pastures with ≤50 animals, the precision of the count was likely high. As pastures were geographically separated, we considered them independent samples.

### Aerial Imagery

Aerial imagery of the CV and BC sites was collected between 10∶44 AM and 3∶07 PM using an airborne remote sensing system consisting of three Kodak Megaplus 4.2i digital cameras, each recording a specific spectral region: green (0.54–0.56 µm), red (0.66–0.68 µm), and near-infrared (0.7–0.9 µm) with a spatial resolution of 25 cm [39]. Imagery was acquired twice for each pasture. We selected images for analysis that were at or near nadir, had less than ≤50 animals, and had reliable ground counts. Across the eight pastures examined, the minimum time between the first image (T1) and the second image (T2) was 48 minutes and the maximum time difference was 148 minutes. Image acquisition likely did not affect animal movements since the aircraft flew at a mean elevation of 549 m above ground level [28], [31].

### Known Animal Locations in Imagery

In order to compare our image difference animal count to actual ground counts, we identified locations for all animals on each pasture image. To do this, we digitally overlaid the two temporal images (T1 and T2) and visually identified changes due to animal movements. These manual animal counts were compared to the number of animals identified in the ground counts, and discrepancies reconciled so that the image counts matched the ground counts. This provided a metric by which we could calculate the accuracy of our semi-automated technique.

Knowledge of the specific locations of each animal allowed us to classify the results of the image differencing into three categories: “mapped” consisted of all image differences identified as animals through the semi-automated approach, “correctly mapped” consisted of image differences that accurately depicted animals, and “incorrectly mapped” consisted of image differences not coincident with a known animal location. We considered any known animal location not associated with an image difference as a “missed animal.” Knowledge of the number of correctly or incorrectly mapped and missed animals allowed us to measure directly errors of commission, identifying a feature as an animal when it was not, and omission, excluding a known animal feature. For our research, we defined the percent of correctly identified animals (P_correct_) as the number of correctly mapped animals divided by number of known animals in the pasture. The percent omission error (P_omiss_) specified missed animals and was calculated as the number of missed animals divided by the number of known animals in the pasture. The percent commission error (P_commiss_) specified incorrectly mapped animals and was calculated by dividing the number of incorrectly mapped animals by the total number of mapped animals in the pasture. The total number of features mapped divided by the number of known animals in a pasture combines errors of commission, omission, and correctly identified animals and was therefore not examined.

### Image Analysis

We radiometrically calibrated images to percent reflectance using an Exotech four-band radiometer nested with the camera system and vignette errors removed using established lens parameters prior to analysis [40]. Rectification of images to the Universal Transverse Mercator System (UTM), NAD83 datum occurred in ERDAS Imagine 9.1.0 using existing ortho-corrected 1 m resolution color imagery collected by the National Agricultural Imagery Program (NAIP). The average root mean square error (RMSE) was 2.0 pixels across all pastures with a range of 0.6 to 4.8 pixels between pastures and the NAIP base map. We considered this RMSE error acceptable since our imagery was collected at a spatial resolution of 0.25 m and the NAIP base map consisted of 1 m pixels. In addition to image to map registration, temporal image-to-image registration spatially linked the T1 image to the T2 image for each pasture by manually connecting features common to both images (i.e. tie points) [41]. The mean image-to-image RMSE was 1.9 pixels across all pastures with a range of 1.1 to 3.1 using an average of 37 ground control points for each pasture [41]. No active farm equipment was present in any of the pastures during image acquisition thus animals were the only features that moved between acquisitions.

**Figure 1:**
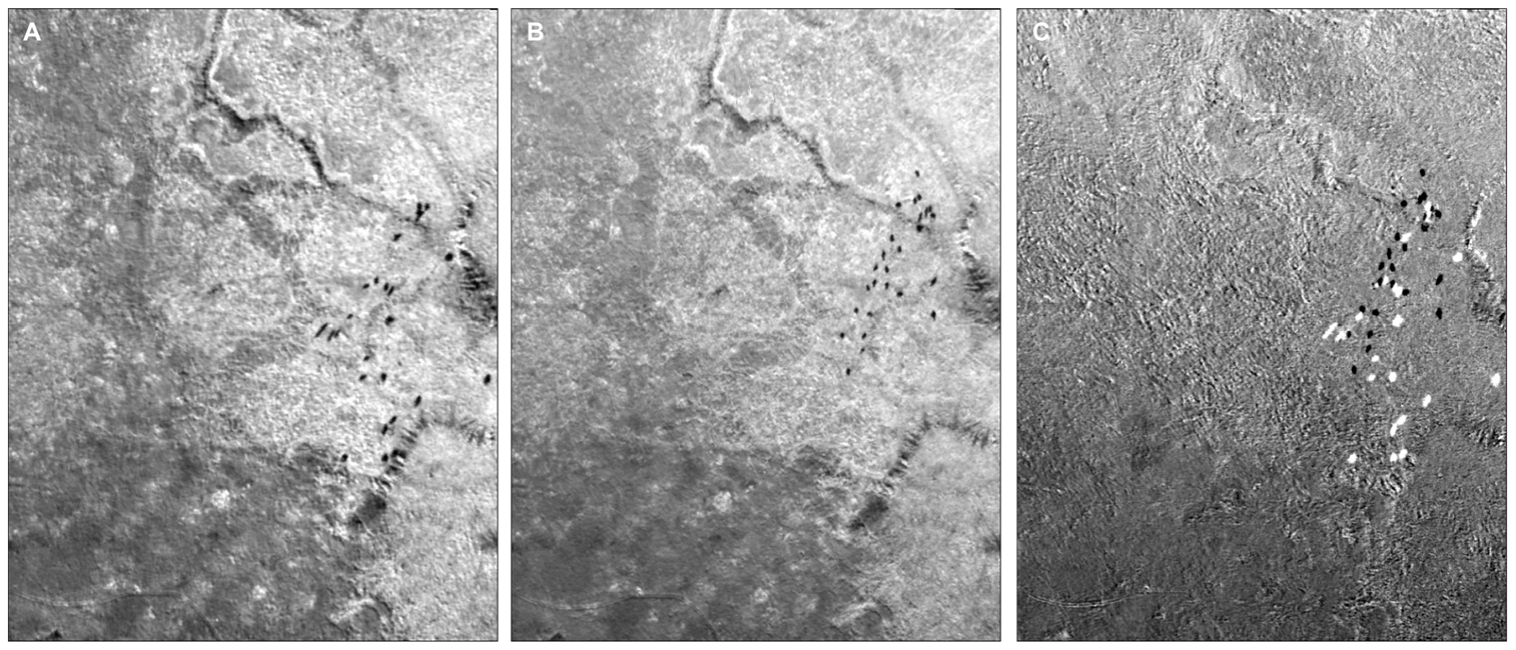
Section of pasture 29 depicting 22 known animals. Figure A is the 1^st^ principal component of the first image acquired (T1), figure B is the 1^st^ principal component of the second image acquired (T2), and figure C is the differenced image resulting from subtracting T1 from T2.

A principal component analysis was conducted on each pasture image to reduce dimensionality and to extract the first component, which contained the highest amount of correlated information between the three input bands [41], [42]. A differenced image was obtained by subtracting the first principal component of the T1 image from the first principal component T2 image (Figure 1). To reduce edge effects, we clipped the differenced images to the minimum extent of T1 and T2. The initial difference image consisted of absolute pixel-to-pixel difference values with low difference values representing inherent image differences caused by atmospheric and/or sensor calibration differences and high difference values representing potential animals. Using the known animal locations from the pasture images, we identified image differences that were correctly associated with animals to establish spectral thresholds that represented animal pixels. Pixels within the spectral threshold values (high difference) were converted to polygons in ArcGIS 10.2 without any smoothing of polygon boundaries thus maintaining size and shape characteristics. Pixels outside the spectral threshold values (low difference) were removed from the analysis. A potential source of error at this step was the inclusion of spectral values from non-animal “edge” pixels into the threshold range representing animals (i.e. pixel or point spread function, [41], [43]. The amount of spectral information incorporated from the pixels surrounding animals was variable and resulted in an unpredictable number of pixels being added to animal features and causing large differences in the size of polygons representing animals. Although the physical size of an adult cow or horse was set as the initial area limit (B. Bowmen, personnel communication), the inconsistencies of polygon sizes associated with known animals forced us to empirically establish a size threshold. Through this process, we determined areas >10 m^2^ were too large to be animals and areas < 0.99 m^2^ were too small to be animals. The 10 m^2^ upper limit was established to account for multiple animals close enough in proximity to be identified as a single feature. Reducing the upper limit below 10 m^2^ had little effect on commission errors but substantially increased omission errors. Polygons outside of the spatial threshold range were removed and the remaining polygons were considered potential animals.

## Results

The average size of the pastures was 6.2 hectares ±2.6 (±STD) with the largest pasture, 9.7 hectares and the smallest pasture 1.9 hectares. The total number of known animals in the eight pastures was 158 with a minimum of three animals in pasture eight and a maximum of 38 animals in pastures seven and two (Table 1). There were 385 total polygons mapped across the eight pastures and per pasture counts ranged from 15 polygons to 136 polygons. The total number of incorrectly mapped polygons was 265 with a mean of 33 (±43) and a minimum of zero polygons in pasture seven and a maximum of 122 incorrectly mapped polygons in pasture six (Table 1).

**Table 1:** The percent correct (P_correct_), the percent omission (P_omiss_), and the percent of commission (P_commiss_) for counting animals from a differencing process between two images acquired on a single day.

Pasture	Known number of animals in pasture	Mapped polygons	Correctly mapped polygons	Polygons representing 2 animals	Missed animals	Incorrectly mapped polygons	P_correct_^1^	P_omiss_^2^	P_commiss_^ 3^
1	18	15	9	0	9	6	50	50	40
2	38	26	22	3	13	4	66	34	15
3	4	10	3	0	1	7	75	25	70
4	29	33	22	1	6	11	79	21	33
5	13	71	12	0	1	59	92	8	83
6	15	136	14	0	1	122	93	7	90
7	38	35	35	2	1	0	97	3	0
8	3	59	3	0	0	56	100	0	95
Sum	158	385	120	6	32	265	-	-	-
Mean	20	48	15	1	4	33	82	18	53
STD	14	41	11	1	5	43	17	17	36

^1.^ (Correctly mapped polygons a/Known number of animals in pasture).

^2.^ (Missed Animals/Known number of animals in pasture).

^3.^ (Incorrectly mapped polygons/Number of mapped polygons).

Animal presence in an image had a high probability of being correctly identified by single day image differencing, but the percent commission error (over-counting) varied considerably among pastures. The mean P_correct_ across the eight pastures was 82% (±17 (STD, Table 1) and the mean P_commiss_ was 53% (±36%), and ranged from 0% to 95%. The mean P_omiss_ was 18% (±17%) and ranged from 0% to 50% (Table 1). The P_correct_ and P_omiss_ were inversely related to each other due to the equation to calculate them, thus as P_correct_ increased, P_omiss_ decreased.

Across the eight pastures, image differencing resulted in six polygons representing multiple individuals (i.e., adjacent animals). One pasture had a single polygon representing two animals, one pasture had two polygons representing four individuals, and one pasture had three polygons representing six individuals (Table 1).

## Discussion

Regardless of the feature to be examined or the temporal frequency, change detection with remotely sensed imagery requires precise spatial registration and correction/normalization of atmospheric differences to avoid false positives [21], [41], [44], [45]. Small misalignments between the T1 and T2 images were identified as changes and resulted in high commission errors. To assess mis-registration, we measured the coordinates of five common features between T1 and T2 for each pasture. The points were different from those used as control points in the image-to-image rectification. All of the pastures had mis-registrations errors less than 1 m in the X direction and 2.11 m in the Y direction (Table 2). The mean Y mis-registration error of 1.06 m could effectively encompass the length of a small adult cow, which suggests small spatial errors could be incorrectly mapped as animals emphasizing the importance of precise image registration when attempting to identify small features with image differencing.

**Table 2:** Mean image-to-image mis-registration errors (STD, standard deviation of five distance differences (meters) for each pasture; SE, standard error) across 5 points in the X and Y directions for eight pastures.

Pasture	Mean X	STD X	Mean Y	STD Y
6	26	14	128	99
1	37	19	59	23
2	40	35	108	88
5	54	50	99	65
7	55	34	35	18
4	70	40	63	63
8	90	39	143	123
3	91	42	211	165
Mean	58		106	
SE	20		37	

Image thresholding is often an exploratory process that requires human interpretation, cannot be replicated, and is often inconsistent [46]–[50]. We attempted to automate the identification of spectral and spatial thresholding values for animal identification in aerial imagery without success [51]. Attributing non-animal pixels to animal features due to the point spread function added to the confusion of identifying specific spatial thresholding values and increased the maximum size of a polygon representing a single animal. While our maximum spatial threshold of 10 m^2^ is arguably large for representation of a single animal, it effectively represented two animals in close proximity.

In addition to mis-registration errors adding to the commission error, shadows also contributed to over-counting errors. Shadows present in images collected on the same day but at different times will exhibit changes, which could mimic animal movements. Images collected at a similar time of day on successive days and if possible, when the sun is directly over-head should reduce shadow effects. The number of days separating T1 and T2 image acquisitions should be less than a week to avoid changes in sun angle and/or dramatic changes in the surrounding vegetation due to growth, senescence, etc. Additionally, 1–2 days, with 7 days maximum, separating image acquisitions should ensure both spatial and temporal population closure so that differences in the number of animals are minimal. To ensure closure of the population, imagery should not be acquired during times of migration or dispersal of young, nor when there is predictable movement of individuals into (i.e., immigration) or out of (i.e., emigration) the population. Additions of newborn animals to the population should be minimal for most species except in spring. Unless imagery acquisition occurs during a hunting season or during a catastrophic die-off, deaths should be minimal between 1–7 days.

In summary, fine scale temporal image differencing will correctly detect 82% of the animals present in an image but also over-count 53%. Thus, certain precautions should be addressed prior to applying this technique. First, although P_omiss_ was relatively low, P_commiss_ was high and was similar to counts in remotely sensed imagery for Canada geese, snow geese, and caribou [52] that were over-estimated due to inclusion of erroneously classified background areas. Second, identification of spectral thresholds that represented animals was a heuristic process that relied on human interpretation, which may not be without bias. Third, image differencing requires precise image registration to avoid spurious areas of change that can result in large numbers of incorrectly mapped polygons. To help mitigate registration errors, ground control points should be set prior to image acquisition to facilitate accurate and precise registration of T1 to T2. Fourth, enough time must pass for animal movement to occur between image acquisitions but not enough time for the population to experience immigration, emigration, births, or deaths. Additionally, species should not be experiencing seasonal or annual migrations. Fifth, the non-animal portions of the image (i.e., the background) should be as homogenous as possible to enhance differentiation between animals and their background. Although there are variations in snow-pack, a snow-covered surface could provide a relatively uniform background.

### Implications to Enumerating Wild Ungulates

Aerial surveys are one method commonly used to estimate the population sizes of ungulates and consist of counting the number of individuals observed on a transect, within a designated strip width, or across a specified area (e.g., 14,18,19, 28–30, 53, 55–56,58). Generally, an assumption is made that not all animals present on the survey were observed and a correction factor is applied to the survey count. The probability of detection adjusts a survey count based on the ratio of the number of animals counted to the number of animals available to be counted during a wildlife survey [53]. One method of determining the number of available animals is to tag (e.g. radio collar, color collar, ear tag) a certain number of animals prior to the survey and record the number of tagged animals observed during the survey. For example, if 20 deer were fitted with color collars prior to an aerial survey and during the survey, 10 of those collared deer were observed, the probability of detection for deer during the survey would be 10/20 or 50%. The count obtained during a survey is then corrected by dividing the count by the probability of detection. Thus, in the above example, if 40 deer were counted on the survey, the adjusted population estimate would be 40/0.50 or 80 deer. Reported values of the probability of detection for conventional wildlife surveys range from 52% in caribou (*Rangifer* spp., [54]), 53–71% for feral ungulate species [55], and 34–82% for mule deer (*Odocoileus hemionus*, [56]) depending on group size and habitat type. Reported detection probabilities of bison (*Bison bison bison*) are higher (>92%) than other wildlife species regardless of habitat or season [57], [58]. If we consider an image a “survey”, our P_correct_, coupled with our P_commission_, is somewhat analogous to the probability of detection in that it reports the correct number of animals identified. Our mean P_correct_ of 82% is near or above reported levels for wildlife surveys and suggests image differencing could provide an alternative method for counting animals across a landscape. Application of our method would require an intense analysis of a small sub-sample of the study area to ascertain the number of known animals in the area and to determine the percent of correctly identified animals and errors of omission and commission. The number of animal located in this area could be enumerated via ground counts or by examining the imagery to determine movements.

Although less than 2% (6 out of 385) of the polygons generated in our process represented two individuals, multiple individuals in close proximity to each other resulted in confusion during the identification of spatial thresholds. Multi-animal polygons could pose an additional complication in application to wildlife species especially when they are in vast herds such as the annual African wildebeest (*Connochaetes taurinus*) migration. Identifying clusters that represent multiple wild animals will require further research and until it is solved, applications of this technique to herding species should recognize the potential under-counting bias for individuals in close proximity to each other.

Drawbacks to wildlife aerial surveys are that errors of omission (not observing an animal that is present during a survey) and commission (animals counted twice) are not generally measured, although see [51]. Knowing the locations of each animal present in the image allowed us to determine errors of omission and commission, which could be incorporated into a single correction factor to adjust counts. Such a correction factor could incorporate the percent correctly identified, the error of omission, and the error of commission. Our sample size was small and of limited scope which prevented the development and evaluation of a correction factor. Additional test cases are needed to develop a correction factor that has practical application.

Satellite imagery could provide an alternative source of imagery, especially in isolated areas such as the Mongolian steppe or large areas such as the western desert of Utah. There are at least six satellites that are currently capable of collecting sub-meter imagery (Quickbird, IKONOS, GeoEye 1, WorldView 1 and 2, and Pleiades 1 and 2) and have the potential to identify large animals. The reduction in time required to acquire satellite imagery over a large study area could facilitate population estimates over areas previously too large or too isolated to survey. Additionally, acquisition of remote sensing aerial or satellite imagery has the potential to reduce or even eliminate negative responses of animal to low flying aircraft during aerial wildlife surveys [28]–[32].
